# Secretory pathway antagonism by calicivirus homologues of Norwalk virus nonstructural protein p22 is restricted to noroviruses

**DOI:** 10.1186/1743-422X-9-181

**Published:** 2012-09-03

**Authors:** Sue E Crawford, Nadim J Ajami, Frederick H Neill, Robert L Atmar, Kazuhiko Katayama, Budi Utama, Mary K Estes

**Affiliations:** 1 Department of Molecular Virology and Microbiology, Baylor College of Medicine, Houston, TX, 77030, USA; 2 Virology II, National Institute of Infectious Diseases, Musashi-murayama, Tokyo, Japan; 3 Present address: Dengue Branch, Centers for Disease Control and Prevention, San Juan, PR, USA

**Keywords:** Calicivirus, Norovirus, p22, Secretory pathway

## Abstract

**Background:**

Our previous report that the Norwalk virus nonstructural protein p22 is an antagonist of the cellular secretory pathway suggests a new aspect of norovirus/host interaction. To explore conservation of function of this highly divergent calicivirus protein, we examined the effects of p22 homologues from four human and two murine noroviruses, and feline calicivirus on the secretory pathway.

**Findings:**

All human noroviruses examined induced Golgi disruption and inhibited protein secretion, with the genogroup II.4 Houston virus being the most potent antagonist. Genogroup II.6 viruses have a conserved mutation in the mimic of an Endoplasmic Reticulum export signal (MERES) motif that is highly conserved in human norovirus homologues of p22 and is critical for secretory pathway antagonism, and these viruses had reduced levels of Golgi disruption and inhibition of protein secretion. p22 homologues from both persistent and nonpersistent strains of murine norovirus induced Golgi disruption, but only mildly inhibited cellular protein secretion. Feline calicivirus p30 did not induce Golgi disruption or inhibit cellular protein secretion.

**Conclusions:**

These differences confirm a norovirus-specific effect on host cell secretory pathway antagonism by homologues of p22, which may affect viral replication and/or cellular pathogenesis.

## Findings

The *Caliciviridae* consists of five genera of (+)ssRNA viruses. The genus *Norovirus* contains the human noroviruses (NoV) that are the predominant cause of gastroenteritis in the US [1] and globally [2]. *Norovirus* is divided into five genogroups (GI-GV) and most human NoV fall within GI and GII [3]. Genogroups are further subdivided into genotypes; genotype GII.4 NoV are currently the most frequently detected in humans [4]. Genogroup V contains murine norovirus (MNV), which is lethal to STAT1^−/−^ mice [5]. The genus *Vesivirus* contains feline calicivirus (FCV), a cause of severe respiratory disease in cats.

The nonstructural protein p22 from Norwalk virus (NV), the prototype NoV, encodes a novel and well-conserved motif that mimics a traditional di-acidic ER export signal [6]. This mimic of an Endoplasmic Reticulum (ER) export signal (MERES) motif allows p22 to gain access to COPII vesicles and is necessary but not sufficient to antagonize COPII vesicle trafficking to induce Golgi disassembly and inhibit cellular protein secretion. Since NV, MNV and FCV all induce Golgi disruption [6-8], we compared calicivirus homologues of p22 for the following: sequence similarity, including conserved motifs; cellular distribution and location following expression; ability to induce Golgi disassembly; and inhibition of cellular protein secretion. We examined human NoV homologues of p22, referred to as “p22-like proteins” (p22L), from the GI.1 NV, the GII.4 Houston virus (HOV) [9], the GII.3 Saitama U201 [10], and the GII.6 Cuernavaca 2007 (CNV07) [11]. We also examined MNV p18 from persistent (CR6 [12]) and non-persistent (CW3 [13]) strains (cDNA provided by “Skip” Virgin of Washington University), and FCV p30 (cDNA provided by John Parker of Cornell University), which are all homologues of p22 [14].

**Figure 1:**
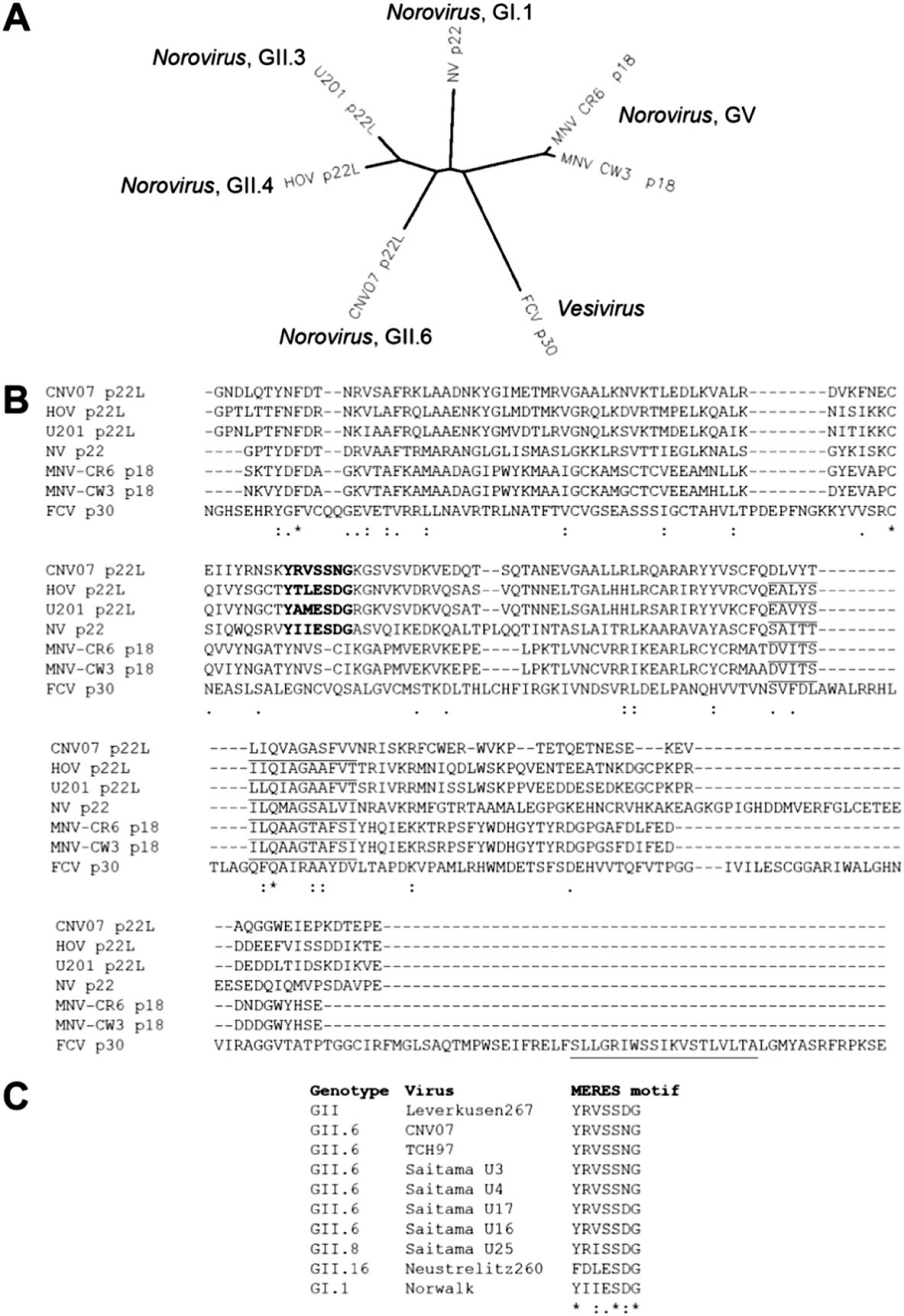
**Relatedness of calicivirus homologues of NV p22.** Calicivirus homologues of NV p22 were compared by phylogeny (**A**) or CLUSTALW amino acid alignment (**B**). CNV07 is Cuernavaca 2007 [GenBank:GU937002], HOV is Houston virus [GenBank:EU310927], U201 is Saitama U201 virus [GenBank:AB039782], NV is Norwalk virus [GenBank:NC_001959], MNV-CR6 and –CW3 are murine norovirus strains CR6 [sequence is identical to [GenBank:EU004676] except for one amino acid change (D50G) (personal communication, “Skip” Virgin)] and CW3 [GenBank:EF014462], respectively, and FCV is feline calicivirus strain Urbana [GenBank:L40021]. Underlined amino acids indicate predicted membrane association domains as predicted by PSIPRED (available online at http://www.psipred.net/psiform.html). Bolded amino acids indicate the MERES motif. (**C**) Amino acids 65–71 of NV p22 were aligned with norovirus p22L that lack full MERES motif conservation. TCH97 is Texas Children’s Hospital 1997 [GenBank:GU930737]; U3 [GenBank:AB039776], U4 [GenBank:AB039777], U16 [GenBank:AB039778], U17 [GenBank:AB039779], U25 [GenBank:AB039780], Neustrelitz260 [GenBank:AY772730] and Leverkusen267 [GenBank:EU424333]. In **B** and **C**, * indicates conserved residues, : indicates strong group conservation, and . indicates weak group conservation.

Phylogeny confirmed distinctions [10] between p22 homologues based on genus, genogroup and genotype (Figure 1A). Amino acid (aa) conservation was ~1% (3 of 275 residues) in caliciviruses and 13% (26 of 201 residues) in human NoV [6]; nonetheless, two conserved motifs were identified. First, an amphipathic α-helix is present in human NoV and MNV p22 homologues corresponding to the membrane association domain (MAD) between aa 112–127 of p22 (Figure 1B). FCV p30 lacks a MAD in this region, but a predicted C terminal amphipathic α-helix may mediate membrane association [15]. Thus, membrane association is likely conserved within all calicivirus homologues of p22. Second, a conserved YXΦESDG MERES motif (where X is any amino acid and Φ is a bulky, hydrophobic residue [e.g. M, I or L]) was present in 92% (65 of 72) of NoV p22L, of which the Y and E residues are most important to secretory pathway antagonism [6]. This motif is absent in MNV p18 and FCV p30.

Of seven identified human NoV that lack the full MERES motif, four belong to GII.6 isolated from different individuals during a single outbreak [10] and encode either YRVSSDG or YRVSSNG in the MERES motif (Figure 1C). The fifth is an untyped GII NoV that also encodes YRVSSDG, the sixth is a GII.8 NoV that encodes YRISSDG, and finally a GII.16 NoV encodes FDLESDG. We sequenced the p22L of two additional GII.6 NoV, CNV07 [11] and TCH97 E99-13646, the latter of which was isolated from an infant male hospitalized with gastroenteritis at Texas Children’s Hospital in 1997. Both viruses encode the same YRVSSNG sequence as other GII.6 NoV, demonstrating retention of the E68S MERES motif mutation in GII.6 NoV from at least 1997 through 2007.

**Figure 2:**
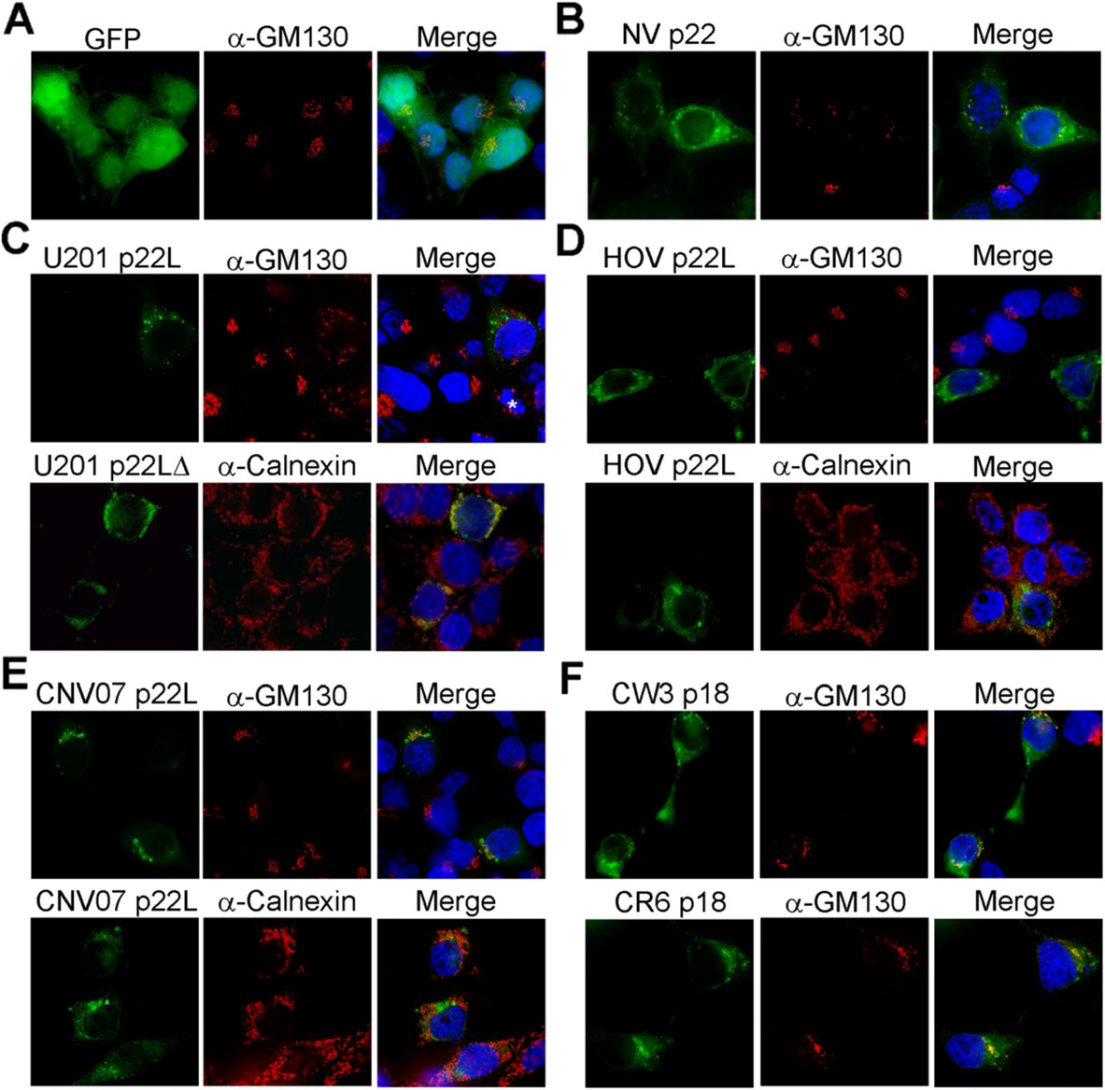
**Localization and effect of norovirus homologues of NV p22 on the Golgi apparatus.** 293T cells were transfected to express the indicated proteins and immuno-stained for the *cis* Golgi or ER marker proteins GM130 and calnexin, respectively (Alexa-Fluor 594 secondary antibody, red fluorescence). Nuclei were counterstained with DAPI (blue fluorescence) and cells were imaged by deconvolution microscopy. The * in **C** indicates a mitotic cell, and “U201 p22LΔ” has the MERES motif mutated to encode YXΦAAAA in place of the MERES motif.

To assess the importance of the MERES motif in secretory pathway antagonism, the cellular distribution and location of N terminal GFP tagged calicivirus homologues of NV p22 and the Golgi phenotype were examined following expression in mammalian cells. At 24 hours post-transfection (hpt), 100% of GFP expressing cells had well-condensed Golgi proximal to nuclei (Figure 2A). Similar to cells expressing NV p22 (Figure 2B) [6], U201 p22L localized to discrete puncta, of which 93% contained phenotypically disrupted Golgi present solely in disordered puncta throughout the cell, similar to Golgi fragmentation in mitotic cells (Figure 2C, top panels, asterisk) [16]. Mutation of the U201 p22L MERES motif to YXΦAAAA resulted in co-localization with the ER marker protein calnexin (Figure 2C, bottom panels), as did p22 containing a similar mutation [6]. This demonstrated a conserved function for the MERES motif in trafficking p22L away from the ER and towards the Golgi. The Golgi in all cells expressing HOV p22L was disrupted (Figure 2D, top panels). HOV p22L localized more diffusely throughout cells without the reticular distribution characteristic of ER localization (Figure 2D, bottom panels) and with few HOV p22L puncta present per cell. Ninety-four percent of cells expressing CNV07 p22L had phenotypically disrupted Golgi, and GFP-tagged protein localized to both the Golgi (Figure 2E, top panels) and ER (Figure 2E, bottom panels), demonstrating an intermediate localization of CNV07 p22L. Similar levels of all p22L by western blot at 24 hpt indicated that differences in Golgi phenotype are not due to differences in protein expression levels (data not shown).

Due to inefficient transfection of macrophages, MNV p18 was expressed in 293T cells to evaluate p18 localization and effect on the Golgi. p18 from MNV strains CW3 and CR6 induced Golgi disruption in 94% and 97% of cells, respectively (Figure 2F). In contrast to p22, MNV p18 puncta co-localized with the peri-nuclear remnants of the Golgi, suggesting that p18 associates with the Golgi despite disruption.

**Figure 3:**
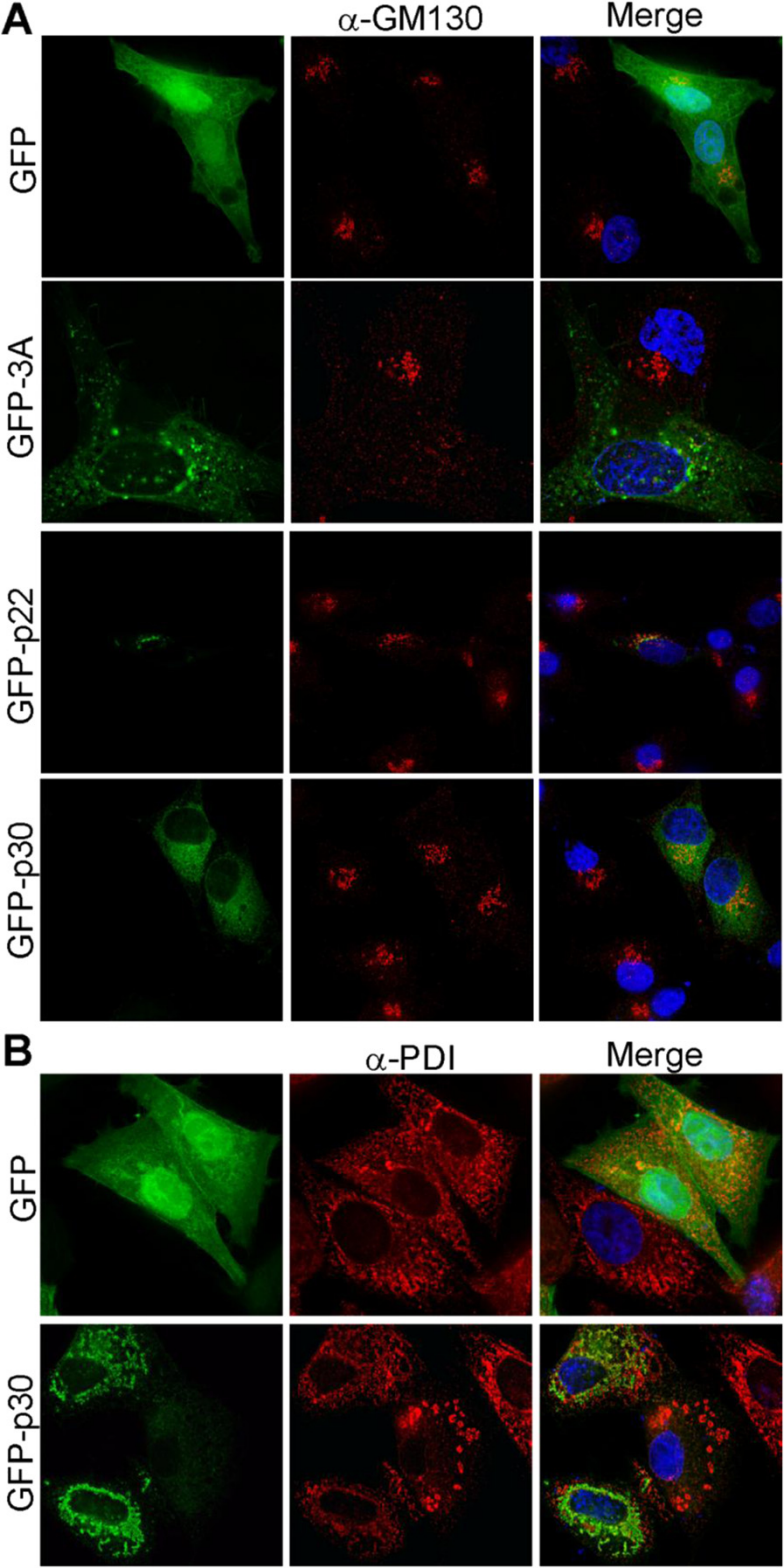
**FCV p30 is ER localized and does not induce Golgi disruption.** CRFK cells were transfected to express GFP or GFP-tagged PV 3A, NV p22 or FCV p30 and immuno-stained (Alexa-Fluor 594 secondary antibody, red fluorescence) for the *cis* Golgi marker protein GM130 (**A**) or the ER marker protein disulfide isomerase (PDI) (**B**). Nuclei were counterstained with DAPI (blue fluorescence) and cells were imaged by deconvolution microscopy.

FCV p30 localizes to and induces ultrastructural changes in the ER and may affect secretory pathway function [15]. Non-transfected or GFP-expressing feline CRFK cells exhibited phenotypically normal Golgi, but the Golgi was dispersed in cells expressing poliovirus (PV) 3A, a well-known secretory pathway antagonist [17-20], and fragmented in cells expressing p22 (Figure 3A). FCV p30 did not induce alterations in Golgi phenotype, but instead exhibited a reticular ER localization (Figure 3B).

**Figure 4:**
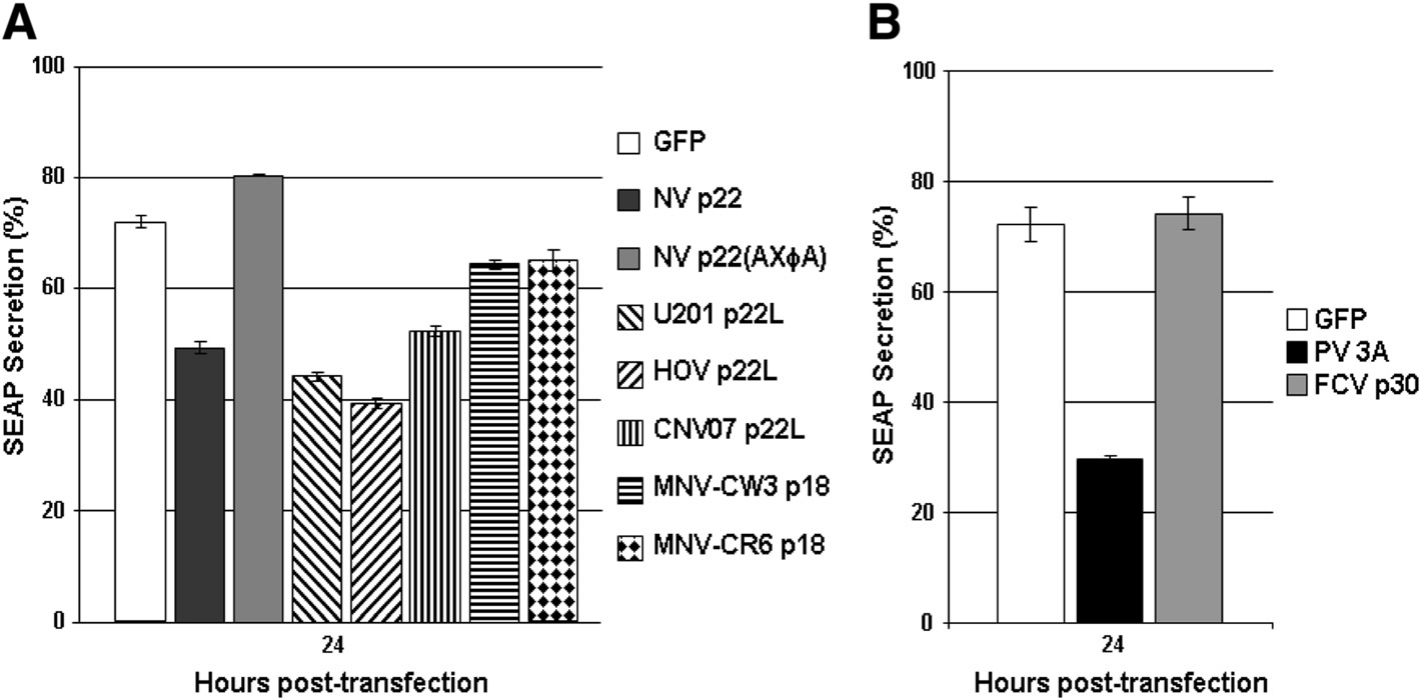
**Effect of calicivirus homologues of NV p22 on cellular protein secretion.** 293T (**A**) or CRFK (**B**) cells were transfected with the vector pCMV-UTR-SEAP expressing the indicated proteins with N terminal GFP tags. Cells were washed and fresh medium was added at 22 hours-post transfection (hpt). At 24 hpt the media (containing extracellular SEAP) was collected, cells were washed 2X in cold 0.01M PBS and lysed in media containing 0.1% Triton X-100 (yielding the intracellular SEAP fraction). SEAP secretion was calculated by assaying both fractions for enzymatic SEAP activity, which were then used in the equation: SEAP secretion (%) = (SEAP_extracellular_/[SEAP_extracellular_ + SEAP_intracellular_]) x 100. Data are representative of at least two experiments; n = 3 for each sample; errors bars indicate standard deviations. One hundred percent of the cells expressing p22 homologue-EGFP and SEAP were viable as assessed by trypan blue exclusion (0.2% final concentration for 5 min) at 24 hpt.

To determine if the observed phenotypic changes in Golgi architecture resulted in changes in secretory pathway trafficking, antagonism of the secretory pathway was examined with the SEAP assay, an enzymatic surrogate for cellular protein secretion [21]. Proteins were expressed from the di-cistronic vector pCMV-UTR-SEAP with N terminal GFP tags and SEAP secretion was quantitated as previously described [6]. At 24 hpt, p22 reduced SEAP secretion to ~50%, representing a 32% reduction in protein secretion compared to GFP alone; mutation of the Y and E residues of the MERES motif in p22 restored SEAP secretion (Figure 4A). p22L from U201 and HOV inhibited SEAP secretion significantly more than NV p22 (p ≤ 0.002) to 44 and 39%, respectively, or 39 and 46% of GFP alone, respectively. Although CNV07 p22L inhibited SEAP secretion by 28% compared to GFP alone, inhibition was significantly less than that of p22 (p = 0.03). Thus, in line with observations of Golgi disruption, HOV p22L was the strongest inhibitor of protein secretion and CNV p22L was the weakest.

p18 from persistent (CR6) and nonpersistent (CW3) MNV strains both inhibited protein secretion significantly less (10% compared to GFP) than p22 (p ≤ 0.0002). FCV p30 did not reduce SEAP secretion in CRFK cells at 24 hpt compared to GFP alone (p = 0.44), although PV 3A efficiently inhibited SEAP secretion (p = 0.00002) (Figure 4B); CRFK cells did not express p22 in sufficient quantity to allow quantitation of SEAP secretion (data not shown).

These results demonstrate that all NoV p22L examined inhibit cellular protein secretion, but to different extents and with differences in cellular localization. Most interesting is the reduction of inhibition seen for CNV07 p22L, which has a natural mutation in the MERES motif. Inhibition of SEAP secretion similarly occurs at intermediate levels when a E68A mutation is made in NV p22 [6], suggesting a causal relationship between this mutation and reduced inhibition of SEAP secretion by CNV07 p22L. Nonetheless, the biological significance of secretory pathway antagonism by NoV p22L, as well as the differences between them in level of inhibition, remains to be elucidated.

Future studies to examine the 20 amino acids conserved between all NoV p22L that are not part of the MERES motif or MAD may assist in identifying the second factor required to mediate secretory pathway antagonism [6]. Additional information may be obtained by comparing NoV p22L with MNV p18, as p18 does not encode the MERES motif but still localizes to the Golgi and disrupts it ([22] and Figure 2). However, lack of the MERES motif in p18 correlates with reduced efficiency of protein secretion inhibition, although Golgi disruption still occurs. This suggests that the MERES motif assists in secretory pathway antagonism by p22L, whereas Golgi disruption is mediated by a region of p22L outside of this motif. This relationship also holds true for FCV p30, which lacks the MERES motif and does not inhibit protein secretion or induce Golgi disruption. Comparison of residues conserved in p22L from NoV and MNV that are absent in FCV will assist in identifying the residues responsible for Golgi disruption.

MNV p18 from persistent and nonpersistent strains had a similar effect on the secretory pathway, suggesting that persistence is not attributable to differences in secretory pathway antagonism by p18. Our result that p18 induces Golgi disruption in human 293T cells is consistent with a report that MNV-infected murine macrophages lack a discernable Golgi [8], and retention of p18 at Golgi remnants suggests antagonism by interaction with a Golgi-localized protein. Indeed, the MNV replication complex is localized peri-Golgi [23], suggesting a role for p18 in its formation.

Since HOV p22L was the most potent secretory pathway antagonist examined, it may be that increased inhibition of protein secretion is beneficial to NoV infectivity or replication. This is supported by the observation that GII.4 p22L undergo positive selection [24]. This coupled with the observation that NV-infected individuals make antibodies specific to p22 ([25] and unpublished observation) suggests that the high variability of p22L may be due to evasion of immuno-recognition.

In summary, secretory pathway antagonism by calicivirus homologues of NV p22 correlates with preservation of the MERES motif, and many p22 homologues affect Golgi phenotype. All homologues examined herein are predicted to be membrane associated, suggesting that despite varying effects on the secretory pathway they may have a conserved role in viral replication. Conservation of alternate and additional functions for p22 homologues should therefore be explored to more fully elucidate how caliciviruses interact with their host cells.

## Abbreviations

Aa: Amino Acids; CNV07: Cuernavaca 2007 Virus; FCV: Feline Calicivirus; HOV: Houston Virus; MAD: Membrane Association Domain; MERES motif: Mimic of an Endoplasmic Reticulum Export Signal; MNV: Murine Norovirus; NoV: Norovirus; NV: Norwalk Virus; p22L: p22-Like protein; PV: Poliovirus; SEAP: Secreted Alkaline Phosphatase.

## Competing interests

The authors declare that they have no competing interests.

## Authors’ contributions

TMS designed and carried out all experimentation and drafted the manuscript. SEC assisted with experimental design and interpretation and manuscript preparation. NJA provided the Cuernavaca 07 virus and assisted in genome sequencing. FN and RLA provided cDNA for Houston virus and isolated TCH97 virus and assisted with genome sequencing. KK provided cDNA for the U201 virus and assisted with experimental design and data analysis. BU assisted with imaging and interpretation. MKE participated in experimental design and interpretation and manuscript preparation. All authors read and approved the final manuscript.
